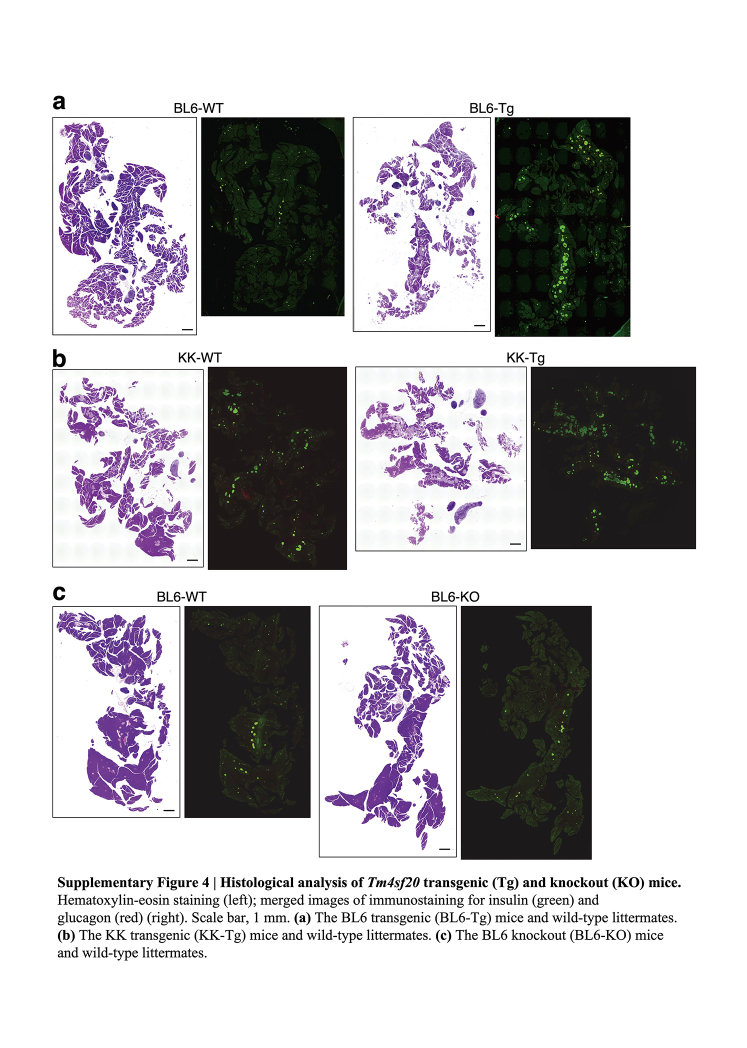# Betagenin ameliorates diabetes by inducing insulin secretion and β-cell proliferation

**DOI:** 10.1016/j.jbc.2025.108202

**Published:** 2025-01-16

**Authors:** Tomotaka Yokoo, Kazuhisa Watanabe, Kaoruko Iida, Yutaka Nakachi, Hiroaki Suzuki, Hitoshi Shimano, Seiji Takashima, Yasushi Okazaki, Nobuhiro Yamada, Hideo Toyoshima

**Affiliations:** 1 Division of Experimental Animal, Hidaka Branch, Biomedical Research Center, Saitama Medical University, Saitama, Japan; 2 Research Center for Genomic Medicine, Saitama Medical University, Saitama, Japan; 3 Department of Endocrinology and Metabolism, Faculty of Medicine, University of Tsukuba, Ibaraki, Japan; 4 Division of Human Genetics, Center for Molecular Medicine, Jichi Medical University, Tochigi, Japan; 5 Department of Food and Nutrition Science, Graduate School of Humanities and Sciences, Ochanomizu University, Tokyo, Japan; 6 Department of Molecular Brain Science, Graduate School of Medical Sciences, Kumamoto University, Kumamoto, Japan; 7 Department of Medical Biochemistry, Osaka University Graduate School of Medicine, Osaka, Japan; 8 Diagnostics and Therapeutics of Intractable Diseases, Intractable Disease Research Center, Graduate School of Medicine, Juntendo University, Tokyo, Japan

**Keywords:** diabetes, pancreatic islet, insulin secretion, cell proliferation, apoptosis

## Abstract

Recent success with the use of glucagon-like peptide-1 (GLP-1) receptor analogs and dipeptidyl peptidase-4 inhibitors for the treatment of patients with diabetes has highlighted the role of the intestine as an endocrine organ. Gut-derived hormones, including glucagon-like peptide-1, glucose-dependent insulinotropic polypeptide, and ghrelin, have important roles in the control of energy metabolism and food intake, and are associated with the metabolic syndrome. In this study, we isolated and identified a new intestine-derived hormone, betagenin, and showed that it stimulates insulin secretion and β-cell proliferation and suppresses β-cell apoptosis. Adenovirus-mediated expression of betagenin restored the blood glucose concentrations and hemoglobin A1c (HbA1c) levels of mice with streptozotocin-induced diabetes to normal and increased their β-cell mass. Transgenic mice overexpressing betagenin exhibited more than three-fold higher β-cell mass than WT mass, whereas that of KO mice was four-fold lower. A synthetic peptide representing the sequence of purified and secreted betagenin enhanced glucose-dependent insulin secretion in human and mouse pancreatic islets and stimulated the proliferation of the pancreatic β-cell line MIN6 through extracellular signal-regulated kinase 1/2-dependent signaling. The intravenous administration of this peptide to streptozotocin mice stimulated the proliferation of pancreatic β-cells *in vivo*, and the intraperitoneal administration of betagenin ameliorated diabetes and restored β-cell mass. These results indicate that betagenin may reduce blood glucose concentration and induce β-cell regeneration in patients with diabetes.

The prevalence of diabetes mellitus (DM) is rapidly increasing, and the disease has become a major healthcare issue worldwide (1). DM is associated with several cardiovascular complications, including coronary artery disease, myocardial infarction, stroke, and peripheral vascular disease, which develop secondary to impaired glucose metabolism. This dysregulation of glucose homeostasis is principally caused by a reduction in insulin secretion by pancreatic β-cells (2), but is exacerbated by a gradual reduction in the mass of functional β-cells, which is a major determinant of the clinical course of DM and the development of comorbidities. Therefore, insulin replacement therapy, which involves daily insulin injections or the administration of drugs that stimulate insulin secretion by β-cells, such as sulfonylureas, is a component of several therapeutic approaches. However, these conventional therapies fail to correct the decrease in the number of functional β-cells (3). Moreover, the overuse of such medication may cause hypoglycemia, which negatively affects the quality of life of patients.

Incretin-related drugs, including glucagon-like peptide-1 (GLP-1) receptor analogs and dipeptidyl peptidase-4 inhibitors, were proposed as treatment options to address these issues in patients with DM (4, 5, 6). Incretins, which are hormones secreted by the intestine, increase insulin secretion by β cells in a glucose-dependent manner. The use of GLP-1 receptor analogs markedly reduces the risk of hypoglycemia, because they do not induce insulin secretion when glucose concentrations are low. In addition, incretin-related drugs may protect β cells, because they have been reported to preserve β-cell mass by suppressing apoptosis (7, 8). Therefore, a drug that would directly increase β-cell mass may represent a potential alternative therapy for patients with DM.

In the present study, a complementary DNA (cDNA) library constructed from RNA isolated from the small intestines of mice was screened for sequences corresponding to secreted proteins using an oligo-cap signal sequence trap (SST) strategy that was developed in our laboratory (9, 10). A combination of *in vitro*, *ex vivo*, and *in vivo* experiments showed that a secreted protein identified in this way, betagenin, is released into the bloodstream and amplifies glucose-dependent insulin secretion and β-cell proliferation through extracellular signal-regulated kinase (ERK) 1/2-dependent signaling. Furthermore, a single administration of synthetic betagenin peptide to mice with diabetes restored their glucose regulation and pancreatic β-cell mass.

## Results

### Identification of betagenin cDNA

**Figure 1:**
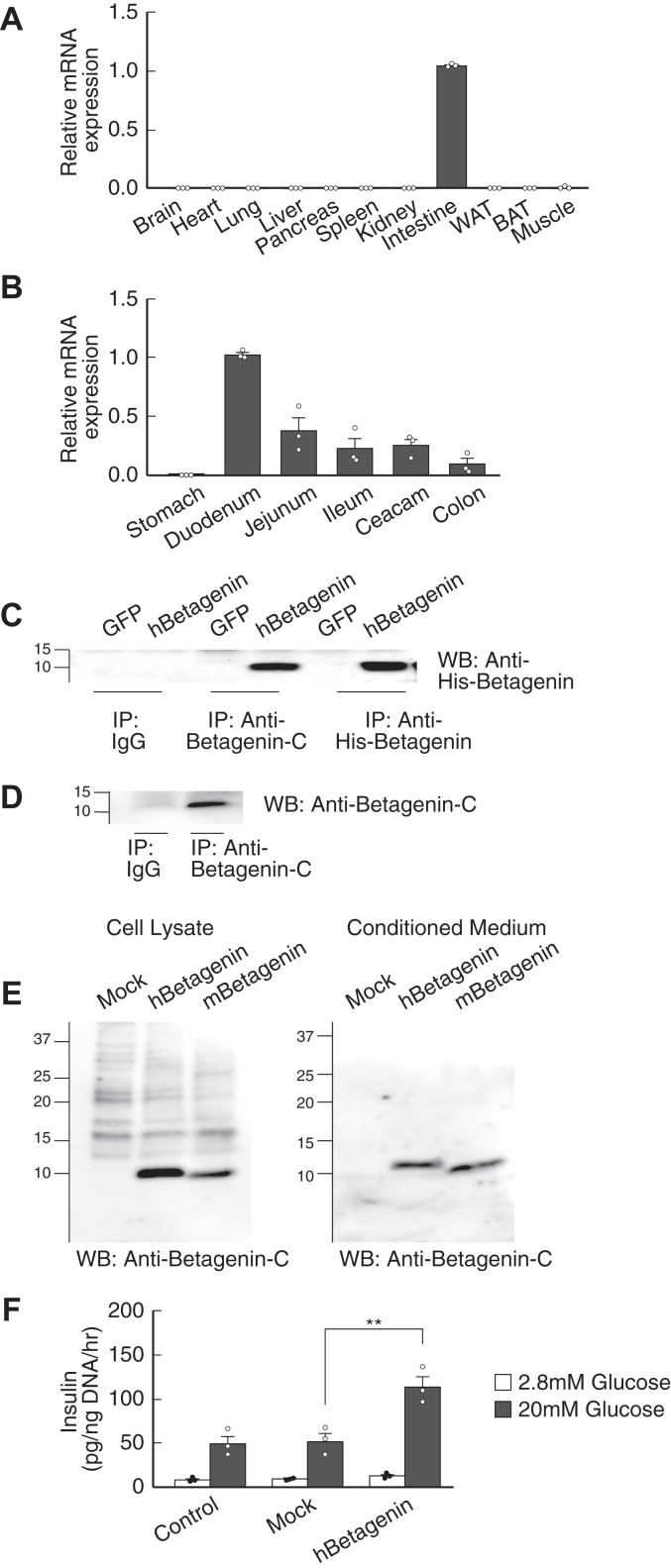
**Betagenin is an intestine-specific secretory protein.***A*, tissue distribution of *Tm4sf20* mRNA, evaluated using qPCR. The values shown are the mean values obtained from three independent experiments. The expression of the target gene was normalized to that of endogenous *Gapdh* expression and the expression in the intestine was arbitrarily set to 1.0 (n = 3). *B*, expression gradient of *Tm4sf20* mRNA in the mouse gastrointestinal tract, evaluated using qPCR, with the expression of *Tm4sf20* in the duodenum being arbitrarily set to 1.0 (n = 3). *C*, Betagenin protein, detected in the blood of BL6 mice infected with adenoviruses expressing human betagenin or GFP using anti-His-betagenin antibody, generated by immunizing rabbits with a His-tagged recombinant protein containing amino acid residues 38 to 226 of betagenin, or anti-betagenin-C antibody, generated by immunizing rabbits with a synthetic peptide representing the C terminus of betagenin, using immunoprecipitation. *D*, endogenous betagenin protein, detected in the blood of BL6 mice using immunoprecipitation. *E*, immunoblotting analysis of betagenin protein expression in cell lysates and medium conditioned by transfected CHO cells. *F*, glucose-stimulated insulin secretion by isolated mouse pancreatic islets (10 islets each, n = 3) exposed to serum-free medium conditioned by HEK293T cells (control, nontransfected cells; mock, cells transfected with pCAGGS; and betagenin, cells transfected with pCAGGS-human betagenin). Data are normalized to the cellular DNA content and are expressed as mean ± standard error. *p*-values were calculated using one-way ANOVA (*F*). ∗∗*p* < 0.01. qPCR, quantitative real-time PCR; Tm4sf20, transmembrane 4 L6 family member 20.

A cDNA clone that was identified in C57BL/6J (BL6) mice using the oligo-cap SST strategy and quantitative real-time PCR (qPCR) analysis was found to encode a gene that was specifically expressed in the intestine (Fig. 1*A*). Furthermore, this gene was expressed at decreasing levels from the duodenum to the colon (Fig. 1*B*). Notably, a previous expression study of incretins in the rat intestine revealed patterns of expression similar to that of glucose-dependent insulinotropic polypeptide (GIP), although in contrast, GLP-1 expression increased toward the colon (11). The sequence of this gene was identical to that of transmembrane 4 L6 family member 20 *(Tm4sf20)*, which encodes a 25-kDa membrane protein of unknown function (12, 13), which we named betagenin. Database comparison (Ensembl Genome Browser) revealed that the predicted amino acid sequence of human betagenin had 75% similarity to that of the product of murine *Tm4sf20*, which is specifically expressed in the small intestine (14). Furthermore, the betagenin gene was found to be highly conserved among mammals (Figs. S1 and S2*A*).

### Betagenin encodes a secreted protein that stimulates insulin secretion

To determine whether the betagenin gene encodes a secreted protein, we studied BL6 mice infected with adenoviruses expressing human betagenin or GFP. Western blot analysis of blood samples from animals infected with adenovirus expressing human betagenin revealed the presence of a 10-kDa protein in immunoprecipitates using two distinct anti-betagenin antibodies: an anti-His-betagenin antibody, prepared by the immunization of rabbits with a His-tagged recombinant protein containing amino acid residues 38 to 226 of betagenin, or an anti-betagenin-C antibody, prepared by the immunization of rabbits with a synthetic peptide representing the C terminus of betagenin (Fig. 1*C*). Similarly, betagenin (10 kDa) was detected in the sera of mice infected with an adenovirus expressing mouse betagenin (not shown). In addition, endogenous betagenin was detected in the bloodstream of WT BL6 mice (Fig. 1*D*). Furthermore, Western blot analysis of whole-cell lysates and the medium used to culture cells transfected with a betagenin expression vector revealed only a 10-kDa band (Fig. 1*E*), corresponding to the molecular mass of the major protein product of the intestinal betagenin gene. Thus, the protein was secreted into the bloodstream and may reach the pancreas.

Using pancreatic islets isolated from BL6 mice, we then investigated the effect of betagenin on insulin secretion. Exposure to medium conditioned by the serum-free culture of HEK293T cells transfected with a betagenin*-*expressing plasmid stimulated glucose-dependent insulin secretion (Fig. 1*F*), implying that the protein encoded by the *betagenin* gene acts similarly to GLP-1 and GIP (4, 5, 6). These findings suggest that the human *betagenin* gene encodes a secreted protein that stimulates insulin secretion by pancreatic β-cells in a glucose-dependent manner.

### Adenoviral expression of betagenin increases the **β**-cell mass and ameliorates the diabetes of streptozotocin-treated mice

**Figure 2:**
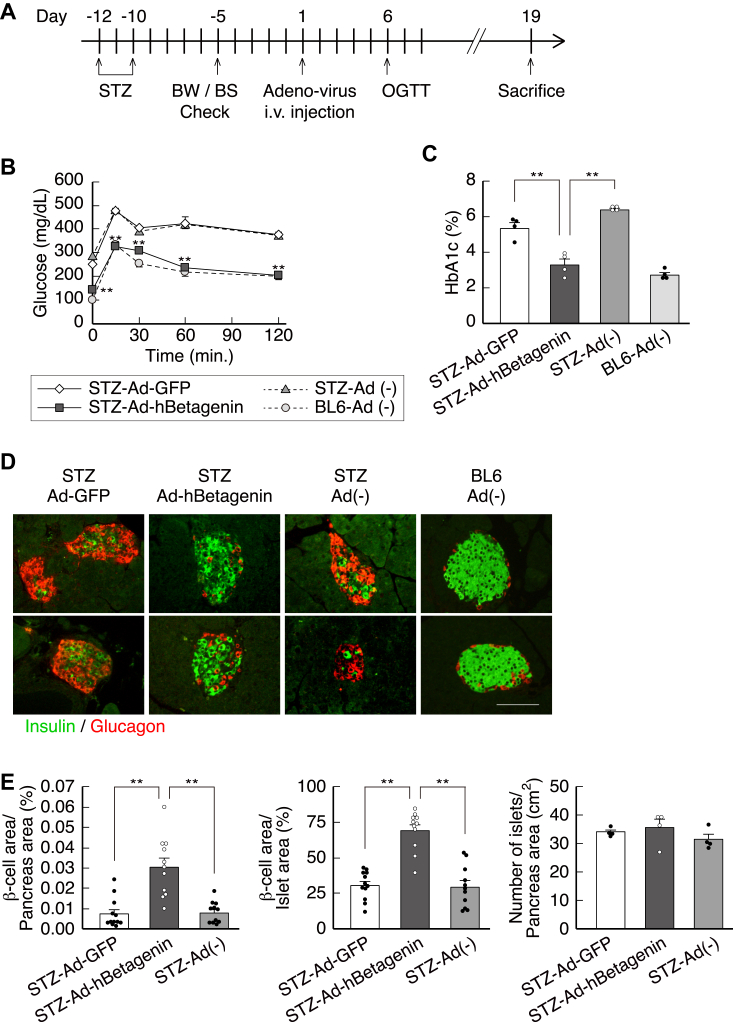
**Adenovirus-mediated expression of human betagenin in mice with STZ-induced diabetes.***A*, days on which injection was performed and on which body mass/blood glucose concentration (BW/BS) was evaluated, and the results of oral glucose tolerance testing. The experiments were conducted in WT BL6 mice (BL6 Ad(−)), STZ BL6 mice (STZ-Ad(−)), and STZ BL6 mice infected with adenoviruses expressing GFP (STZ-GFP) or human betagenin (STZ-hBetagenin) (n = 4 each). *B* and *C*, infection of STZ mice with human betagenin-expressing adenovirus restored their blood glucose concentrations to normal (∗∗*p* < 0.01 *versus* STZ-GFP) and reduced their HbA1c levels (∗*p* < 0.05 *versus* STZ-GFP). *D*, histology of the pancreas. *Green*, insulin immunostaining of β-cells; red, glucagon immunostaining of α-cells. The scale bar represents 50 μm. *E*, β-cell/pancreatic area, β-cell/islet area, and number of islets/pancreatic area ratios were calculated using the sections shown in (*D*) (11–12 islets each). Infection with the human betagenin adenovirus increased β-cell mass three-fold, without affecting the number of islets. ∗∗*p* < 0.01. Data are expressed as mean ± standard error. *p*-values were calculated using one-way ANOVA (*B*, *C*, and *E*). HbA1c, hemoglobin A1c; STZ, streptozotocin.

We next evaluated the therapeutic potential of betagenin *in vivo* using mice with streptozotocin (STZ)-induced diabetes, caused by the destruction of pancreatic β cells (Fig. 2*A*). PBS (STZ-Ad(−)), or adenoviruses expressing human betagenin (STZ-hBetagenin) or GFP (STZ-GFP), were intravenously administered to diabetic mice, and the resulting phenotypes were compared with those of untreated WT (BL6) mice. On day 6, oral glucose tolerance testing (OGTT) revealed that betagenin almost completely reversed the hyperglycemia induced by STZ (Fig. 2*B*). On day 19, the measurement of hemoglobin A1c (HbA1c) level revealed that the glycemic control of STZ-hBetagenin mice was comparable to that of BL6 mice, but significantly better than that of STZ-GFP mice (Fig. 2*C*).

Immunohistochemical analysis showed that the pancreatic islets of BL6 and STZ-hBetagenin mice were primarily populated by β cells (Fig. 2*D*). In contrast, the pancreatic islets of STZ-Ad(−) and STZ-GFP mice predominantly contained glucagon-producing α-cells. Quantitative analysis showed that when compared to STZ-GFP mice, betagenin expression increased the area populated by β-cells in STZ-hBetagenin mice approximately three-fold, without affecting the number of islets (Fig. 2*E*). These results suggest that betagenin improves the glycemic control of diabetic mice by increasing insulin secretion and β-cell mass.

### Betagenin transgenic and KO mice

**Figure 3:**
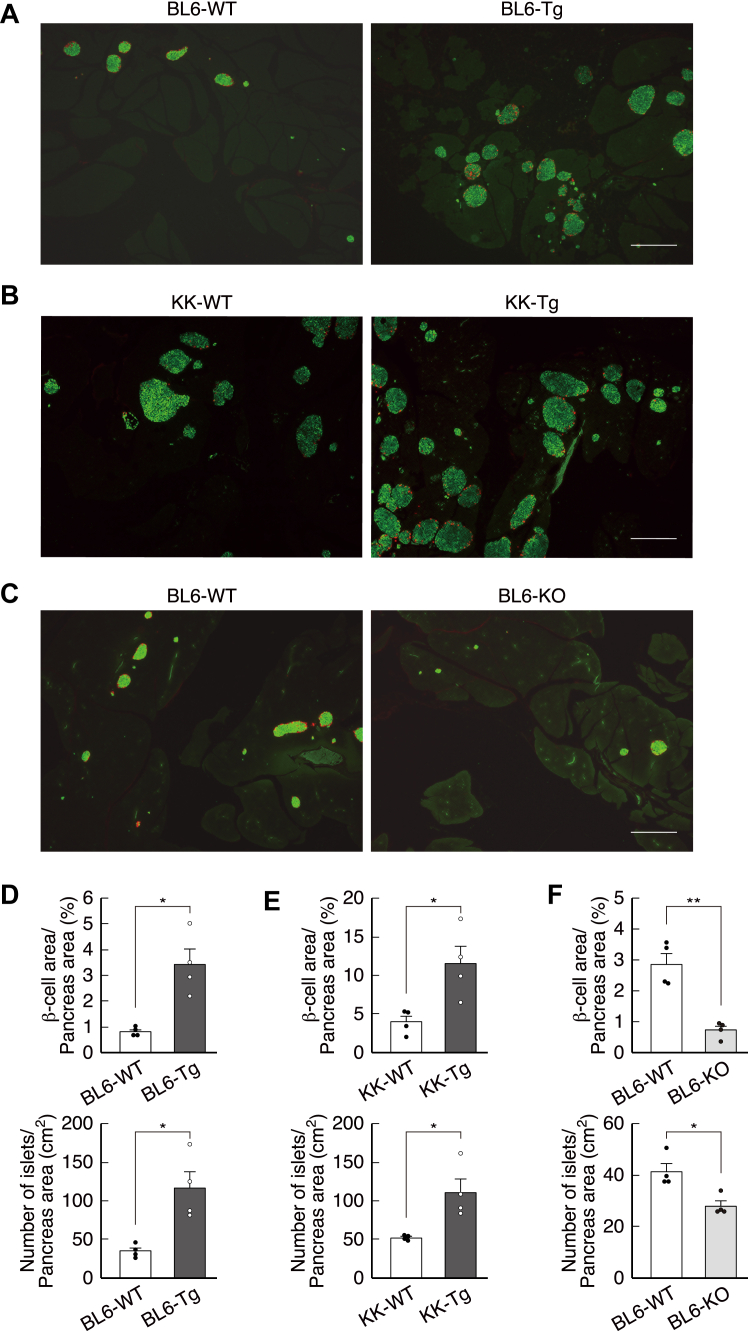
**Histology of the β-cells and islets of betagenin transgenic (Tg) and KO mice**. *A*–*C*, merged images of immunostained sections (insulin, *green*; glucagon, *red* (*right*)). The scale bar represents 1 mm (n = 4 each). *D*–*F*, quantitative analysis of the β-cell area and number of islets across the pancreatic sections shown in a, *B*, and *C*. ∗*p* < 0.05. ∗∗*p* < 0.01. *A* and *D*, BL6-Tg and BL6 mice with the betagenin transgene, (*B* and *E*) KK-Tg and KK mice with the betagenin transgene, (*C* and *F*) BL6-KO and BL6 betagenin KO mice. WT littermates of betagenin Tg or betagenin KO mice. Data are expressed as mean ± standard error. *p*-values were calculated using the unpaired *t* test (*D*–*F*).

Using the BL6 and KK strains, two independent laboratories generated betagenin transgenic mice by directly injecting a betagenin expression construct (Fig. S3, *A* and *B*) into the eggs of each mouse strain. The two transgenic strains appeared to be normal in size and body mass, but the β-cell mass and number of islets were three-fold higher than those of their WT littermates (Figs. 3, *A*, *B*, *D*, and *E* and S4), which is consistent with the effect of betagenin on β-cell mass demonstrated by the infection of mice with betagenin adenovirus.

To determine whether betagenin expression was important for the maintenance of normal pancreatic morphology, we also generated betagenin KO mice (Fig. S3, *C* and *D*). Although these mice appeared to be normal in size, their β-cell/pancreatic area ratios were more than four-fold lower than those of their WT littermates, and they also had significantly fewer islets (Figs. 3, *C* and *F* and S4). These results suggest that intestinal betagenin secretion is essential for the maintenance of a normal pancreatic β-cell population. Mice lacking GLP-1 receptors have been shown to have normal pancreatic morphology, as indicated by the β-cell/α-cell area ratio (15), which suggests the absence of a proliferative effect. Therefore, functional mutations of *betagenin* may predispose individuals toward glucose intolerance.

### Synthetic betagenin peptide induces **β**-cell proliferation

We purified human betagenin from the conditioned medium of cultured cells expressing human *betagenin* transgenes introduced by adenoviral or plasmid expression vectors. We then determined the amino acid sequence of the purified secreted protein and synthesized a betagenin peptide with 60 amino acid residues. Notably, the sequence of this peptide is highly conserved among species (Fig. S2*B*).

**Figure 4:**
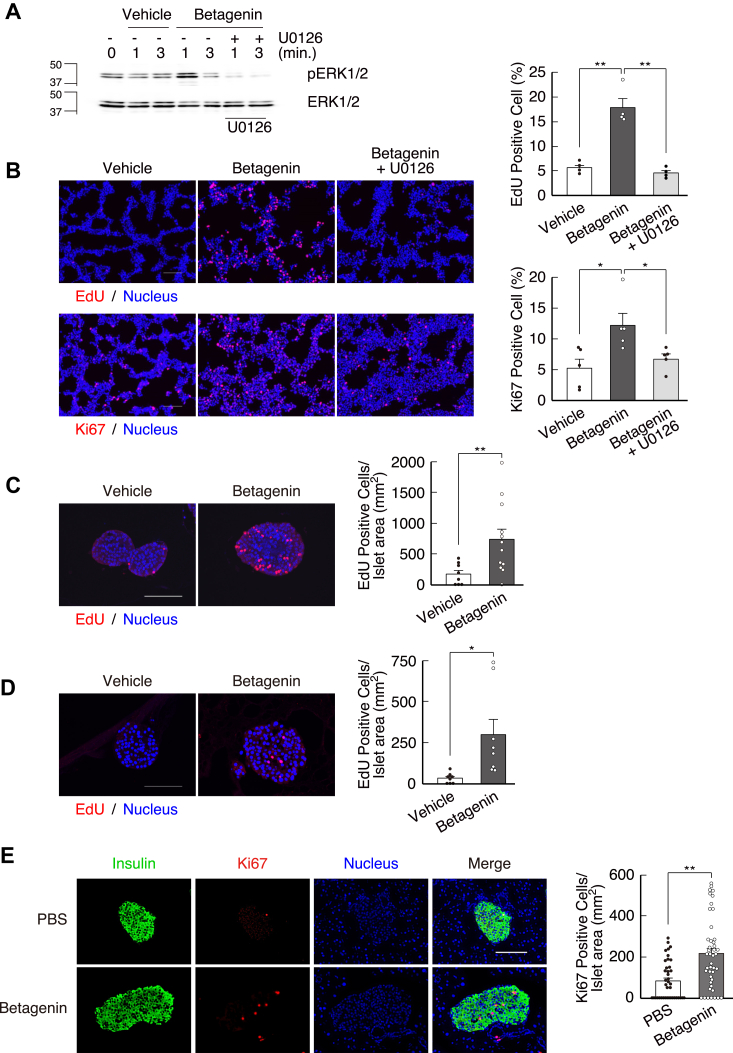
**Synthetic betagenin peptide induces β-cell proliferation**. *A*, the stimulation of ERK1/2 phosphorylation in the MIN6 mouse insulinoma cell line was inhibited by U0126, a MAPK inhibitor. *B*, the betagenin peptide stimulated MIN6 cell proliferation, as indicated by merged images of EdU incorporation (*red*) or Ki67 immunostaining (*red*) with Hoechst 33342 (*blue*). The scale bar represents 200 μm. The percentages of EdU- and Ki67-positive cells were calculated from the images (n = 5) ∗*p* < 0.05, ∗∗*p* < 0.01. *C* and *D*, The betagenin peptide stimulated the proliferation of mouse (*C*) and human (*D*) pancreatic islets, as indicated by the merged images of EdU incorporation (*red*) and Hoechst 33342 (*blue*). The scale bar represents 100 μm. Quantitative analysis of the number of EdU-positive islets over the entire islet area, calculated using the images (n = 8–12) ∗*p* < 0.05. *E*, the betagenin peptide stimulated the proliferation of mouse pancreatic islets *in vivo*, as indicated by insulin immunostaining (*green*), Ki67 immunostaining (*red*), Hoechst 33342 (*blue*), and the merged images. The scale bar represents 100 μm (n = 3 mice). The percentage of Ki67/insulin-positive cells was calculated using the images (n = 40–44 islets). Data are expressed as mean ± standard error. ∗*p* < 0.05, ∗∗*p* < 0.01. *p*-values were calculated using the unpaired *t* test (*C*–*E*) or one-way ANOVA (*B*). EdU, 5-ethynyl-2′-deoxyuridine; ERK, extracellular signal-regulated kinase; MAPK, mitogen-activated protein kinase.

Subsequently, signaling pathways that might mediate the effects of the betagenin peptide were identified using the MIN6 mouse insulinoma cell line. The betagenin peptide induced ERK1/2 phosphorylation in the MIN6 cells, and this was prevented by an inhibitor of mitogen-activated protein kinase (MAPK) (U0126) (Fig. 4*A*). We then showed that the betagenin peptide induced MIN6 cell proliferation, as evidenced by the incorporation of 5-ethynyl-2′-deoxyuridine (EdU) (Fig. 4*B*) and immunohistochemical staining for Ki67 (Fig. 4*B*), both of which were also inhibited by U0126 (Fig. 4*B*). These results are consistent with the central role of 38-, 42-, and 44-kDa MAPKs in MIN6 cell proliferation (16).

We also determined whether the betagenin peptide has similar effects in mouse and human pancreatic islets, finding that the betagenin peptide increased EdU incorporation in both mouse and human islets, indicating a proliferative effect *in vivo* (Fig. 4, *C* and *D*).

Moreover, intravenous injection of the betagenin peptide increased the number of Ki67-positive β cells in BL6 mice three-fold, indicating an induction of β-cell proliferation *in vivo* (Fig. 4*E*).

### Betagenin peptide suppresses **β**-cell apoptosis and induces insulin secretion

**Figure 5:**
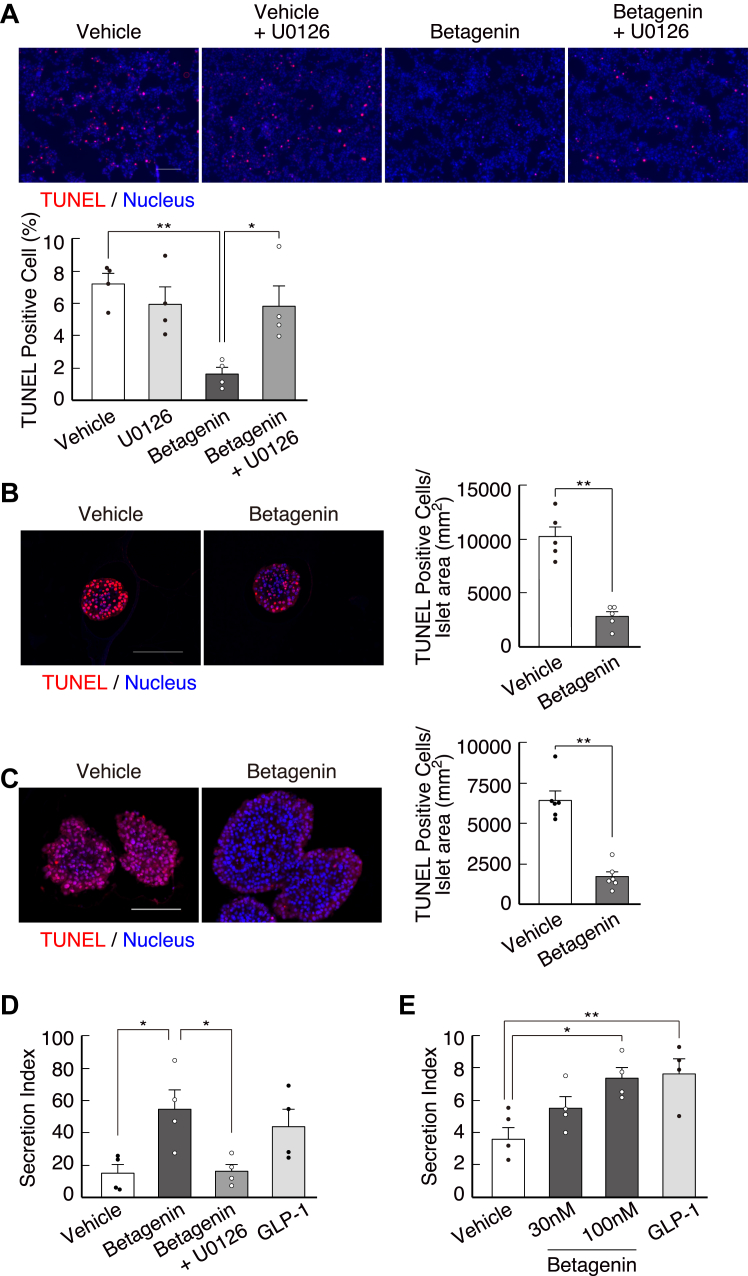
**Synthetic betagenin peptide suppresses β-cell apoptosis and induces insulin secretion**. *A*, the betagenin peptide suppresses serum starvation-induced apoptosis in MIN6 cells, as indicated by the merged images of TUNEL-positive cells (*red*) and Hoechst 33342-stained nuclei (*blue*). The scale bar represents 200 μm. The percentages of TUNEL-positive cells were calculated using the images (n = 4). ∗*p* < 0.05, ∗∗*p* < 0.01. *B* and *C*, the betagenin peptide suppresses apoptosis in serum-starved mouse (*B*) and human (*C*) pancreatic islets, as indicated by the merged images of TUNEL-positive cells (*red*) and Hoechst 33342-stained nuclei (*blue*). The scale bar represents 100 μm. Quantitative analysis of the number of TUNEL-positive islets over the entire islet area using the images (n = 5–6) ∗∗*p* < 0.01. *D* and *E*, glucose-dependent insulin secretion by isolated mouse (*D*) and human (*E*) pancreatic islets. The islets were treated with vehicle (dimethyl sulfoxide), betagenin peptide (1 nM), betagenin and U0126 (10 μM), or GLP-1 (10 nM) (n = 4). Data are normalized to the cellular DNA content. All data are the stimulation index (HG/LG ratio) and expressed as mean ± standard error. *p*-values were calculated using the unpaired *t* test (*B* and *C*) or one-way ANOVA (*A*, *D*, and *E*). ∗*p* < 0.05. GLP-1, glucagon-like peptide-1; HG, high glucose; LG, low glucose.

We then investigated the effect of the betagenin peptide on apoptosis. Serum starvation induced apoptosis in MIN6 cells, but the addition of the betagenin peptide reduced this apoptosis through activation of MAPK pathways (Fig. 5*A*). Similarly, the betagenin peptide suppressed apoptosis in both human and mouse pancreatic islets (Fig. 5, *B* and *C*). We found that this peptide enhanced insulin secretion in mouse and human pancreatic islets in a glucose-dependent manner (Fig. 5, *D* and *E*) and that this response was also inhibited by U0126 (Fig. 5*D*). Similarly, GLP-1 has been shown to have antiapoptotic effects in β-cells that are mediated through ERK1/2 activation (17).

### Betagenin peptide increases **β**-cell mass and ameliorates diabetes in mice with STZ-induced diabetes

**Figure 6:**
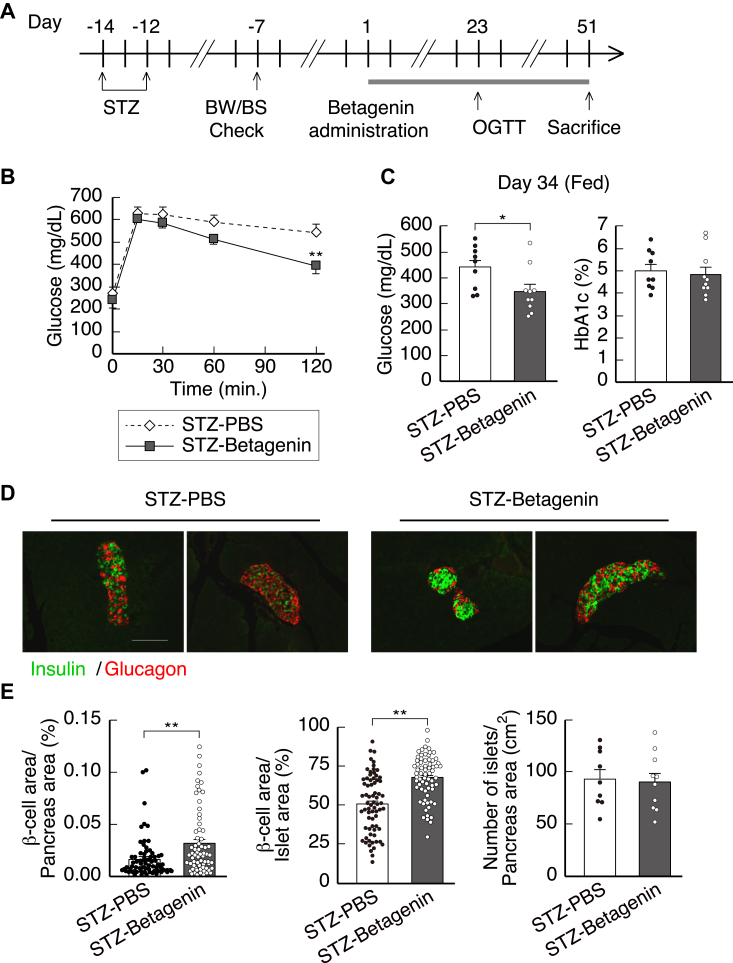
**Long-term effects of a single administration of the synthetic betagenin peptide to mice with STZ-induced diabetes.***A*, days on which injections were performed and on which BW/BS was evaluated, and the results of OGTT. *B*, results of OGTT performed on day 23 (n = 10 each). *C*, postprandial blood glucose concentration and HbA1c level on day 34 (n = 10). *D*, immunostained pancreatic sections: merged images of insulin- (*green*) and glucagon- (*red*) positive cells. The scale bar represents 50 μm. *E*, β-cell/pancreatic area, β-cell/islet area, and number of islets/pancreatic area ratios were calculated using the images in d (n = 68–70 islets). Data are expressed as mean ± standard error. *p*-values were calculated using the unpaired *t* test (*B*, *C*, and *E*). ∗*p* < 0.05, ∗∗*p* < 0.01. HbA1c, hemoglobin A1c; OGTT, oral glucose tolerance testing; STZ, streptozotocin.

We next investigated the long-term effects of the betagenin peptide on diabetes by administering PBS or the peptide (10 μg/mouse; intraperitoneally) twice daily for 51 days to mice with STZ-induced diabetes (Fig. 6*A*). OGTT showed that betagenin improved the glycemic status of the mice (Fig. 6*B*) and reduced their baseline blood glucose concentrations (Fig. 6*C*). However, betagenin did not significantly affect the HbA1c levels of the mice (Fig. 6*C*). Immunohistochemical analysis of the pancreata of the mice showed that the long-term treatment of the diabetic mice with the betagenin peptide caused a significant increase in β-cell mass (Fig. 6, *D* and *E*). These results indicate that a synthetic peptide derived from betagenin improves the glycemic status of mice with STZ-induced diabetes by stimulating insulin secretion and increasing the functional mass of β-cells.

## Discussion

The success of incretin-related drugs as treatments for diabetes highlights the role of the intestine in the production of hormones that regulate metabolism (4, 5, 6). In the present study, betagenin, which is a putative intestinal secreted protein, induces glucose-dependent insulin secretion by pancreatic islets and increases the number of β-cells and islet mass. We also found that a synthetic peptide equivalent to the putative betagenin secreted product improves the glycemic status of mice with STZ-induced diabetes, suggesting that it might be clinically useful.

Several substances have been shown to induce β-cell proliferation (17, 18, 19, 20, 21, 22, 23, 24, 25, 26). An increase in the number of β cells following betagenin treatment was demonstrated by experiments conducted *in vivo*, *ex vivo*, and *in vitro* that included an analysis of the recombinant adenoviral expression of betagenin in mice with STZ-induced diabetes, an assessment of the phenotypes of betagenin transgenic and KO mice, and an evaluation of the biologic activity of a synthetic betagenin peptide. The results suggest that the betagenin peptide stimulates β-cell proliferation by activating MAPK signaling. Furthermore, there were few β cells in the betagenin KO mice, which strongly suggests that betagenin is important for the maintenance of β-cell mass.

Mice with STZ-induced diabetes showed a normalization of blood glucose concentration in response to the adenovirus-induced overexpression of betagenin. However, their β-cell areas remained smaller than those of WT mice. A study of gene expression in the livers of these mice suggested an upregulation of glycolysis and a suppression of gluconeogenesis, which may have been the result of changes in glucose production and uptake in the liver and skeletal muscle. However, betagenin peptide administration had weaker effects in mice than adenovirally mediated overexpression, and therefore the effective dose and modifications of the peptide should be studied in the future.

The synthetic betagenin peptide has incretin-like activity; *i.e.*, it enhances insulin secretion in a glucose-dependent manner (4, 27, 28). Because this effect was inhibited by the MAPK inhibitor U0126, it is likely that the effects of betagenin are mediated through the MAPK pathway. Furthermore, injection of an adenovirus expressing recombinant betagenin or the betagenin synthetic peptide into mice with STZ-induced diabetes reduced their hyperglycemia and increased their β-cell mass, which likely increased insulin secretion.

The betagenin cDNA sequence is identical to that of *Tm4sf20*, which is a putative membrane protein of unknown function. Although a membrane-associated form of betagenin/TM4SF20 may exist, Western blot analysis of cell lysates and medium conditioned by betagenin/TM4SF20-transfected cells yielded a single 10-kDa band, but no larger proteins, which might have represented a membrane-associated form. Furthermore, immunohistochemical analysis using two distinct antibodies did not demonstrate the presence of betagenin/TM4SF20 on the membrane (not shown). Notably, the two antibodies detected bands of the same size on Western blots. Because the 10-kDa band was also identified following the Western blotting of the sera of mice infected with a betagenin-expressing adenovirus, a 10-kDa secreted protein may be the principal endogenous product of the gene. In addition, a small amount of endogenous betagenin was also detected in blood samples obtained from the mice by immunoprecipitation.

Public databases suggest that the protein is expressed not only in the small intestine, but also in the brain, pancreas, and other tissues. However, in several of these databases, mRNA expression is only described in the intestine. When taken together with the results of our qPCR analysis, this suggests that the principal site of expression is the intestine, although it is possible that the protein is expressed in other locations. Wiszniewski *et al.* showed that the absence of a single *TM4SF20* exon is associated with a pediatric syndrome characterized by language delay and white matter hyperintensities (29). Their results suggest that this phenotype is caused by the cytoplasmic accumulation of an abnormally truncated product of betagenin/TM4SF20 that is generated through improper splicing in the central nervous system (CNS). Although the betagenin/*Tm4sf20* expression in the brains of BL6 mice was not assessed using qPCR analysis, betagenin/*Tm4sf20* may play roles in certain cells of the CNS.

Ceramide has been reported to regulate the translocation of TM4SF20 by altering membrane topology (30, 31, 32). Post translocation, this protein cleaves and activates the transcription factor Creb3l1, which is associated with cell proliferation. In the future, the expression of endogenous TM4SF20 should be characterized in terms of timing, localization, molecular weight, and activity. The results may provide insight into the mechanism and details of this cleavage. However, it may also be important to characterize these signals during cell proliferation.

Current antidiabetic drugs, such as those related to incretins, do not induce as much β-cell proliferation, and therefore betagenin may offer advantages for the treatment of patients with either type 1 diabetes or type 2 diabetes (T2DM). β-cell destruction causes type 1 diabetes, and therefore betagenin has the potential to be a first-line treatment for this condition, given that it increases the size of the β-cell population. A partial recovery of β-cell function markedly improves the quality of life of patients with DM. In addition to exogenous insulin, physiologic insulin secretion by the patients’ β cells should contribute to the precise control of blood glucose concentration and reduce the risk of hypoglycemia.

A relative decrease in insulin secretion is the primary cause of T2DM. Betagenin may be useful for the treatment of T2DM, because it increases insulin secretion in a glucose-dependent manner. Similar to incretins and GLP-1 receptor agonists (33), betagenin does not stimulate insulin secretion when the circulating glucose concentration is low, thereby reducing the risk of hypoglycemia. Furthermore, because a small β-cell population is a major contributor to the progression of DM (34), betagenin’s unique ability to increase the number of β cells, and possibly islets, may represent a significant advantage regarding its potential therapeutic use, because it should preserve or lead to the recovery of insulin production, thereby improving the long-term prognosis of patients. Moreover, because β cells have been reported to be capable of proliferating while in their terminally differentiated state (35, 36), betagenin could also be used to increase the size of β-cell populations *in vitro*.

We have shown that betagenin affects the number of pancreatic islets, directly or indirectly, possibly by affecting the proliferation, as well as the differentiation, of islet-cell populations. Therefore, along with its potential use to induce the differentiation of pluripotent stem cells, betagenin represents a promising potential regeneration therapy for patients with DM (37).

GLP-1 and GIP regulate glucagon secretion by α cells and may affect appetite and gut motility *via* the CNS (4, 6, 33). Further research is warranted to determine whether betagenin has such effects. In addition, the identification of the betagenin receptor will be an important aspect of further studies of the physiologic roles of betagenin.

The most important limitation of the present study is that the physiologic significance of betagenin is unknown, and the role of this molecule has not yet been fully characterized. To address this limitation, the mechanisms governing betagenin expression, translation, and cleavage must be clarified. Knowledge regarding the timing of its expression and under what dietary conditions it is released into the circulation is critical. In addition, the specific receptors must be identified and the associated signaling pathway must be characterized. Knowledge of the signal transduction pathways associated with betagenin is important to elucidate the mechanisms of its effects on β-cell proliferation and insulin secretion. Currently, drugs that increase insulin secretion are in clinical use, and attempts to increase pancreatic β-cell numbers are being studied but are unfinished. Because of its effects, betagenin may represent a target for the identification of new diabetes therapies. In addition, the development of an assay system to measure the circulating concentrations of betagenin would help to clarify the relationships of betagenin with diet and diabetes.

In conclusion, we have identified a novel intestinal secreted factor that regulates insulin secretion and β-cell proliferation. The identification of betagenin’s unique ability to induce β-cell regeneration may lead to the development of new and more effective therapeutic strategies for diabetes.

## Experimental procedures

### Materials

Murine interleukin-3 (IL-3) was obtained from R&D Systems. Polyclonal antibodies against mouse betagenin were produced by immunizing rabbits with either a His-tagged recombinant protein consisting of amino acid residues 38 to 226 of betagenin (anti-His-betagenin) or a 13-amino acid synthetic peptide representing the C terminus of betagenin (anti-betagenin-C). Both antibodies readily detected their respective mouse and human homologs. Antibodies against p44/42 MAPK (Erk1/2) and phospho-p44/42 MAPK (Erk1/2) (Thr202/Tyr204) were obtained from Cell Signaling Technology.

### Animals

Eight-week-old male C57BL/6J mice were purchased from CLEA Japan and permitted to adapt to their new environment for 1 week before the initiation of the study. The mice were randomly assigned to an experimental group, and housed in a controlled environment under a 12-h light/dark cycle, with free access to water and standard laboratory diet. Diabetes was induced by two intraperitoneal injections of STZ (100 mg/kg body mass, Sigma-Aldrich), administered 1 day apart. The STZ was dissolved in 50 mmol/l sodium citrate buffer (pH 4.5) immediately before administration. The mice were considered to have become diabetic when their blood glucose concentrations exceeded 300 mg/dl, which was usually within 7 days of the first injection (38, 39).

The animal housing and all the animal experimental protocols were approved by the Animal Experiment Committee of the Saitama Medical University and University of Tsukuba (approval number: 3676).

### Generation of betagenin transgenic mice

Betagenin transgenic mice were generated at the Laboratory Animal Resource Center, University of Tsukuba (BL6 background) and CLEA Japan (KK background). To generate the mouse *Tm4sf20* transgenic mice, cDNAs encoding the CAG promoter, mouse *Tm4sf20* (amino acids 1–226), and the 3′ polyadenylation signal of rabbit β-globin were microinjected into the ova of C57BL6J or KK mice (Fig. S3*A*). A PCR-based assay that distinguished the three possible genotypes confirmed gene integration. Founder mice (BL6 or KK strains) were backcrossed at least six times with mice of the same genetic background, then heterozygous mice were intercrossed to obtain transgenic and WT siblings.

### Generation of betagenin KO mice

Betagenin KO mice were created by Kurabo Industries. After confirming the integration site of the neomycin cassette between exons 1 and 3 (Fig. S3*C*), we developed a PCR-based assay to distinguish the three possible genotypes. Betagenin KO founder mice (BA1 hybrid; 50% 129SvEv, and 50% C57BL, six strains) were backcrossed at least six times to transfer the null mutation onto the BL6 genetic background.

### Cloning of the betagenin cDNA using the oligo-cap SST strategy

After standard feeding, followed by 12 h of fasting, mRNA was extracted from the small intestines of BL6 mice using a FastTrack2.0 mRNA isolation kit (Invitrogen, Thermo Fisher Scientific), according to the manufacturer’s instructions. cDNA fragments were synthesized using a FirstChoice RLM-RACE Kit (Ambion, Thermo Fisher Scientific) and then ligated into the retroviral pMX-SST vector (a kind gift of Dr Toshio Kitamura, University of Tokyo) (40). The resulting library consisted of 2 × 10^6^ independent clones with cDNA insert sizes ranging from 0.3 to 2 kb. Using the Plat-E retroviral packaging cell line (a gift of Dr T Kitamura), Ba/F3 cells were infected with high-titer retroviruses containing a mouse small intestinal cDNA library. The integrated cDNA fragments were isolated from IL-3-independent Ba/F3 clones using genomic PCR (40). All the cDNA fragments were sequenced and analyzed. This cloning system has been described in detail previously (9, 10).

### Constructs

Reverse transcription–PCR was used to obtain mouse betagenin/*Tm4sf20* cDNA. The plasmid used to express full-length betagenin (pCAGGS-betagenin) was constructed by ligating this cDNA into the pCAGGS vector, which contains the CAG promoter (41). In addition, a plasmid expressing His-tagged betagenin (amino acid residues 38–226, lacking the signal sequence) was constructed by ligating the cDNA into the pET28a vector (Novagen).

### RNA isolation and quantitative real-time PCR

RNA was isolated from tissue using TRIzol (Invitrogen), according to the manufacturer’s instructions. We quantified *Tm4sf20* and *Gapdh* mRNA expression by qPCR analysis, as previously reported (42). The template used for the qPCR analysis was prepared using ReverTra Ace qPCR RT Master Mix with gDNA Remover (Toyobo Bio) using the extracted RNA. We then performed qPCR for the genes of interest using cDNA-specific TaqMan primer/probe sets (TaqMan Gene Expression Assays, Applied Biosystems, Thermo Fisher Scientific) on a StepOnePlus Real-Time PCR system (Applied Biosystems). The mouse TaqMan probe/primer sets used were as follows: *Tm4sf20* (ID; Mm04208575_m1) and *Gapdh* (ID; Mm99999915_g1; used as the reference gene) (Applied Biosystems). Gene expression was calculated using the 2^−ΔΔCt^ method and is presented as a difference from the matched experimental control mice.

### Cell culture and transfection

MIN6 cells (a gift of J. Miyazaki) were maintained in Dulbecco’s modified Eagle’s medium (DMEM, Gibco, Thermo Fisher Scientific) supplemented with 15% fetal bovine serum (FBS, Sigma-Aldrich), 1% penicillin/streptomycin (Gibco), and 71.5 μM β-mercaptoethanol (Gibco) (43, 44). Plat-E, HEK293T, and 293A cells (Invitrogen) were maintained in DMEM supplemented with 10% FBS and 1% penicillin/streptomycin. Ba/F3 cells were maintained in RPMI-1640 medium supplemented with 10% FBS, 10 ng/ml mouse IL-3, and 1% penicillin/streptomycin. CHO-K1 cells, obtained from the RIKEN Bioresource Center, were maintained in Ham’s F12 Nutrient Mixture (Gibco) supplemented with 10% FBS and 1% penicillin/streptomycin. For transfections, 5 × 10^5^ cells were grown in 100-mm culture dishes and transfected using FuGENE6 (Roche), according to the manufacturer’s instructions (45).

### Immunoprecipitation and immunoblot analysis

Immunoblotting of plasma and cell lysates and the immunoprecipitation analysis of plasma samples were conducted as previously reported (42, 44, 46, 47). Briefly, cells were homogenized in lysis buffer [1% Triton X-100, 0.5% NP-40, 10% glycerol, 0.45% sodium pyrophosphate, 100 mM NaF, 1 mM Na_3_VO_4_, protease inhibitor cocktail (Roche), 9.2 mM Hepes (pH 8.0), and 11.5 mM NaCl] and centrifuged at 18,000*g* for 20 min at 4 °C and the supernatants (whole-cell lysates) were collected. Mouse serum samples were fractionated by ammonium sulfate precipitation. Proteins precipitated using 50% saturated ammonium sulfate were removed, and those precipitated using 70% saturated ammonium sulfate were dissolved in PBS and then used for immunoprecipitation. Whole-cell lysates and fractionated serum samples were subjected to immunoprecipitation using a rabbit polyclonal anti-His-betagenin or anti-betagenin-C antibody and protein G-Sepharose beads (GE HealthCare). After an 1-h incubation at 4 °C, the beads were washed three times in lysis buffer and then boiled briefly in SDS-PAGE sample buffer to elute the proteins for subsequent electrophoresis. The proteins were separated on 12% SDS-PAGE gels, transferred onto polyvinylidene fluoride membrane (Immobilon-P, Millipore), blocked with 5% skimmed milk or 5% bovine serum albumin (BSA) in Tris-buffered saline (20 mM Tris–HCl pH 7.5 and 100 mM NaCl) containing 0.1% Tween-20 (TBS-T). Betagenin, Erk1/2, and phospho-Erk1/2 were detected using 1:2000 dilutions of anti-His-betagenin or anti-betagenin-C, anti-Erk1/2, and anti-phospho-Erk1/2 antibodies, respectively, in Tris-buffered saline containing 0.1% Tween-20. Bound antibodies were detected using a horseradish peroxidase-coupled anti-rabbit IgG secondary antibody (Cell Signaling Technology) and visualized using enhanced chemiluminescence substrates (GE HealthCare). The chemiluminescence was detected using an ImageQuant LAS 4000 system (GE HealthCare).

### Isolation of mouse pancreatic islets

Pancreatic islets were isolated from BL6 mice using the Ficoll–Conray protocol (44, 48, 49). Briefly, the animals were euthanized, the common bile duct of each was clamped close to its opening into the duodenum, 4 mg/ml of collagenase (Sigma-Aldrich) was injected into the pancreatic duct, and the expanded pancreas was excised. After 20 min of incubation at 37 °C, the islets were purified on a Ficoll gradient, then the islets were cultured for 2 h in RPMI-1640 medium supplemented with 10% FBS at 37 °C in a humidified atmosphere containing 5% CO_2_. To evaluate islet proliferation, the mouse islets were cultured for 2 days in 10 μM EdU ± the betagenin peptide. The cultured mouse islets were then formalin-fixed, paraffin-embedded, and sectioned.

### Human islet preparation

Nondiabetic human islets were purchased from Prodo Labs, in accordance with their rules for ethical sample procurement. In total, 10 human islets were embedded in collagen gel (Nitta Gelatin) and cultured directly in Prodo Islet Medium (PIM(R)) for 2 days at 37 °C in a humidified atmosphere containing 5% CO_2_, and then an insulin secretion assay was performed. To evaluate islet proliferation, human islets were cultured in PIM(S) (Prodo labs) containing 10 μM EdU ± the betagenin peptide for 7 days. For the apoptosis assay, the human islets were cultured for 12 h.

### Assay of insulin secretion by pancreatic islets

Insulin secretion by isolated islets was measured as previously reported (44, 48, 49). Briefly, groups of 10 islets with similar sizes were preincubated in Krebs–Ringer bicarbonate buffer (KRBH, pH 7.4: 10 mM Hepes, 129.4 mM NaCl, 5.2 mM KCl, 2.7 mM CaCl_2_, 1.3 mM KH_2_PO_4_, 1.3 mM MgSO_4_, and 24.8 mM NaHCO_3_) containing 0.5% BSA and 2.8 mM glucose. They were then exposed to serum-free medium conditioned by betagenin-transfected HEK293T cells (30%) or betagenin synthetic peptide (1 nM) in KRBH containing 0.5% BSA and 2.8 mM glucose (low glucose) for 30 min, followed by the same medium containing 20 mM glucose (high glucose) for 30 min. A Mouse Insulin ELISA Kit (Shibayagi) or a Human Insulin ELISA Kit (Mercodia) was used to measure the insulin concentrations of the media. Hoechst 33258 (Sigma-Aldrich) was used to determine the DNA contents of sonicated islets. The amount of insulin secreted at a specific high glucose concentration was divided by the amount secreted at a specific low glucose concentration (2.8 mM), and then the secretion index was calculated.

### Preparation of recombinant adenovirus and transduction

Full-length Betagenin cDNA and the CAG promoter were subcloned into the pENTR4 vector (Invitrogen) through homologous recombination with the pAd promoterless vector (Invitrogen) to create a recombinant adenoviral plasmid. Recombinant adenoviruses were generated in 293A cells and purified using CsCl gradient centrifugation (50, 51). Intravenous injections of adenoviruses (1 × 10^8^ PFU) expressing GFP or betagenin were then administered to the mice.

### Oral glucose tolerance testing

The mice injected with adenoviruses were fasted for 12 h, then glucose (1 g/kg body mass) was orally administered, and the plasma glucose and insulin concentrations of the mice were measured at various time points (50, 51).

### Metabolism measurements

Plasma glucose and insulin concentrations were measured as previously reported (51). Glucose concentrations were measured using a Glucose Oxidase kit (Wako), and insulin concentrations were measured using a Mouse Insulin ELISA kit (Shibayagi) or a Human Insulin ELISA Kit (Mercodia). An HbA1c immunoassay kit (DCA2000 system; Siemens) was used to measure the HbA1c level (48).

### Immunohistochemistry

Formalin-fixed, paraffin-embedded pancreatic samples were sectioned and immunohistochemically stained to analyze insulin expression. The sections were immunostained with guinea pig anti-insulin (Dako, Agilent Technologies) and rabbit anti-glucagon (Dako) antibodies after dewaxing in xylene and rehydration. To assess β-cell proliferation, MIN6 cells were immunostained with rabbit anti-Ki67 antibody (Abcam), and expression was detected using fluorescein-conjugated secondary antibodies (Paris, France and Rockland Immunochemicals). The sections were also incubated for 5 min with 0.01% Hoechst 33342 dye (Sigma-Aldrich) to stain the nuclei (38, 48). All the islets on the sections were evaluated using fluorescence microscopy (BZ-X700; Keyence), and the proportions of the pancreatic sections or pancreatic islets that were occupied by β cells were calculated using BZ-X Analyzer software (Keyence) (17, 20, 52). Ki67 expression was also used to measure the proportion of immunostained nuclei (17, 20, 52).

### Incorporation of EdU

EdU incorporation was quantified, as an index of DNA synthesis, to assess the effect of betagenin on β-cell proliferation *in vitro*. MIN6 cells were starved for 72 h in DMEM supplemented with only 0.5% FBS and incubated for 22 h in the presence or absence of 1 nM betagenin peptide in DMEM containing 0.5% FBS. After 2 h of incubation with EdU (10 μM), the incorporated EdU was detected using a Click-iT EdU Alexa Fluor 594 Imaging Kit (Life Technologies, Thermo Fisher Scientific). A BZ-X700 microscope was used to acquire images, and BZ-X Analyzer software was used to calculate the number of cells that incorporated EdU (mitotic cells) (17, 20, 52).

### TUNEL assay

A TUNEL assay was performed using a Click-iT Plus TUNEL Assay for In Situ Apoptosis Detection Kit (Life Technologies). MIN6 cells were serum-starved for 72 h in DMEM supplemented with only 0.5% FBS ± 1 nM betagenin peptide. Mouse islets were serum-starved for 4 days in RPMI1640 supplemented with only 0.1% FBS. Human islets were cultured for 12 h. Cultured human or mouse islets were then formalin-fixed, paraffin-embedded, and sectioned. Apoptotic cells were stained according to the manufacturer’s instructions, and a BZ-X700 microscope was used to acquire images.

### Injection of the betagenin synthetic peptide

Betagenin peptide (1.5 mg/kg body mass) or PBS was injected into tail veins of BL6 mice, and then the proliferative effect of betagenin *in vivo* was evaluated 2 days later. Mice with STZ-induced diabetes were used to assess the effect of betagenin on diabetes *in vivo* (38). These mice were intraperitoneally injected with PBS (control) or betagenin peptide (10 μg/mouse) twice a day for 51 days.

### Statistical analysis

Data are expressed as mean ± standard error. We performed each experiment in at least triplicate to confirm the reproducibility of the data obtained. Datasets were compared using the unpaired *t* test or unpaired one-way ANOVA to compare the means of two and more than two groups, respectively. All analyses were performed using the software package R (R Foundation for Statistical Computing, Vienna, Austria, https://cran.r-project.org/). *p* < 0.05 was considered to indicate statistical significance.

## Data availability

All the data that support the findings of this study are available from the corresponding author upon reasonable request.

## Supporting information

This article contains supporting information.

## Conflict of interest

The authors declare that they have no conflicts of interest with the contents of this article.

## Figures and Tables

**Figure S4:**