# Supplementary material for: Betagenin ameliorates diabetes by inducing insulin secretion and β-cell proliferation
Source: J Biol Chem. 2025 Jan 16;301(2):108202. doi: 10.1016/j.jbc.2025.108202 (PMC11870162; doi:10.1016/j.jbc.2025.108202)
Supplement: Figure S1 [file mmc1.pdf]

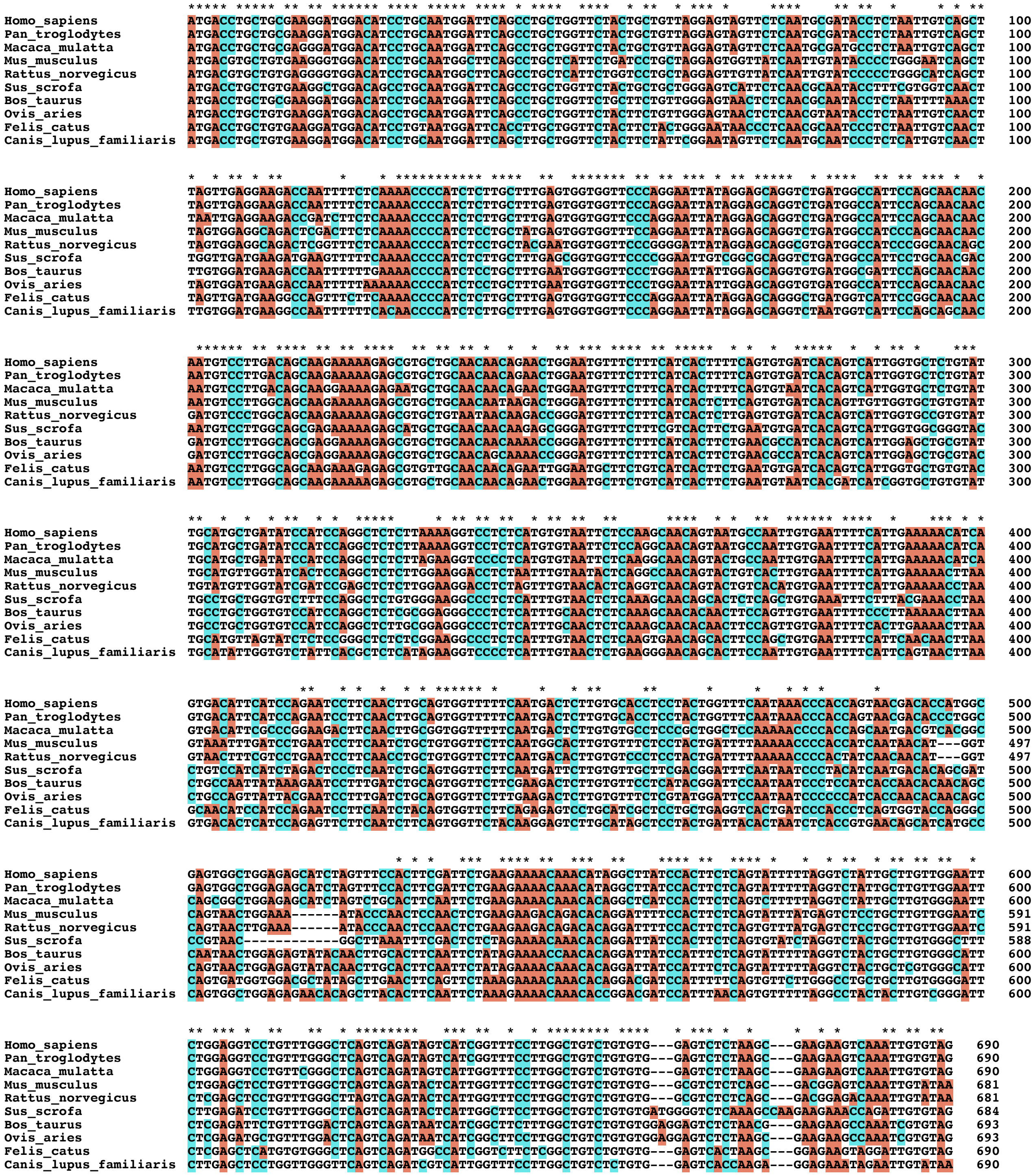

Supplementary Figure 1 | The *TM4SF20* gene is highly conserved.

The nucleic acid sequences of *TM4SF20* homologs was aligned using Clustal X software.
