# Supplementary material for: Betagenin ameliorates diabetes by inducing insulin secretion and β-cell proliferation
Source: J Biol Chem. 2025 Jan 16;301(2):108202. doi: 10.1016/j.jbc.2025.108202 (PMC11870162; doi:10.1016/j.jbc.2025.108202)
Supplement: Figure S2 [file mmc2.pdf]

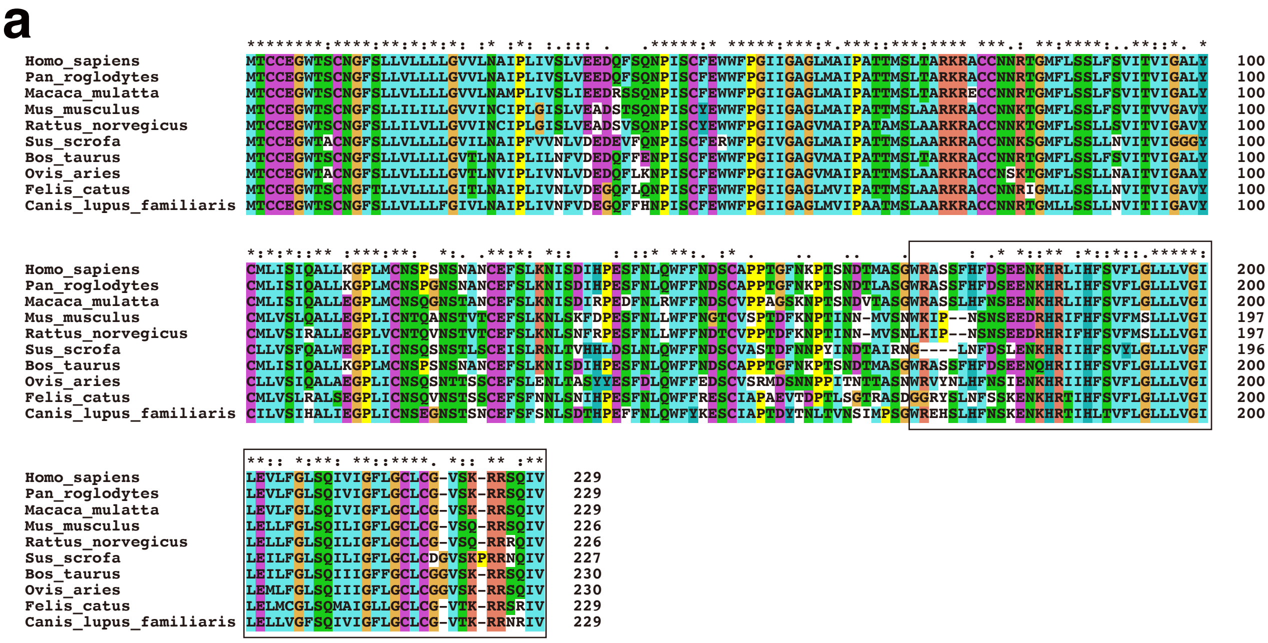

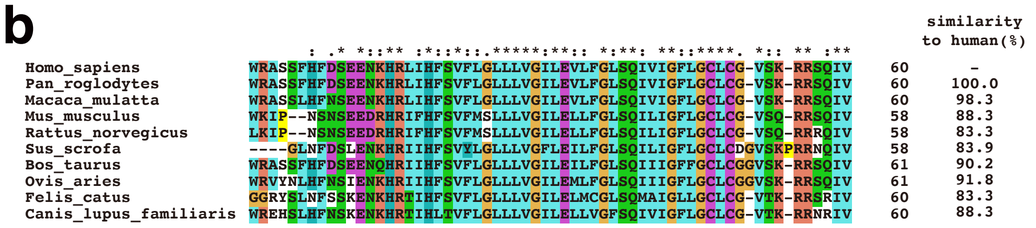

Supplementary Figure 2 | The TM4SF20 amino acid sequence.
The amino acid sequence alignment of **(a)** full-length TM4SF20 and **(b)** the 60 putative amino acid synthetic betagenin peptide with homologs using Clustal X software. In **a**, the box indicates the sequence of the synthetic peptide **(b)**. In **b**, the percentage similarity to human TM4SF20 was calculated using the EMBOSS Needle program (BLOSUM62 was used as matrix).
