# Supplementary material for: Betagenin ameliorates diabetes by inducing insulin secretion and β-cell proliferation
Source: J Biol Chem. 2025 Jan 16;301(2):108202. doi: 10.1016/j.jbc.2025.108202 (PMC11870162; doi:10.1016/j.jbc.2025.108202)
Supplement: Figure S3 [file mmc3.pdf]

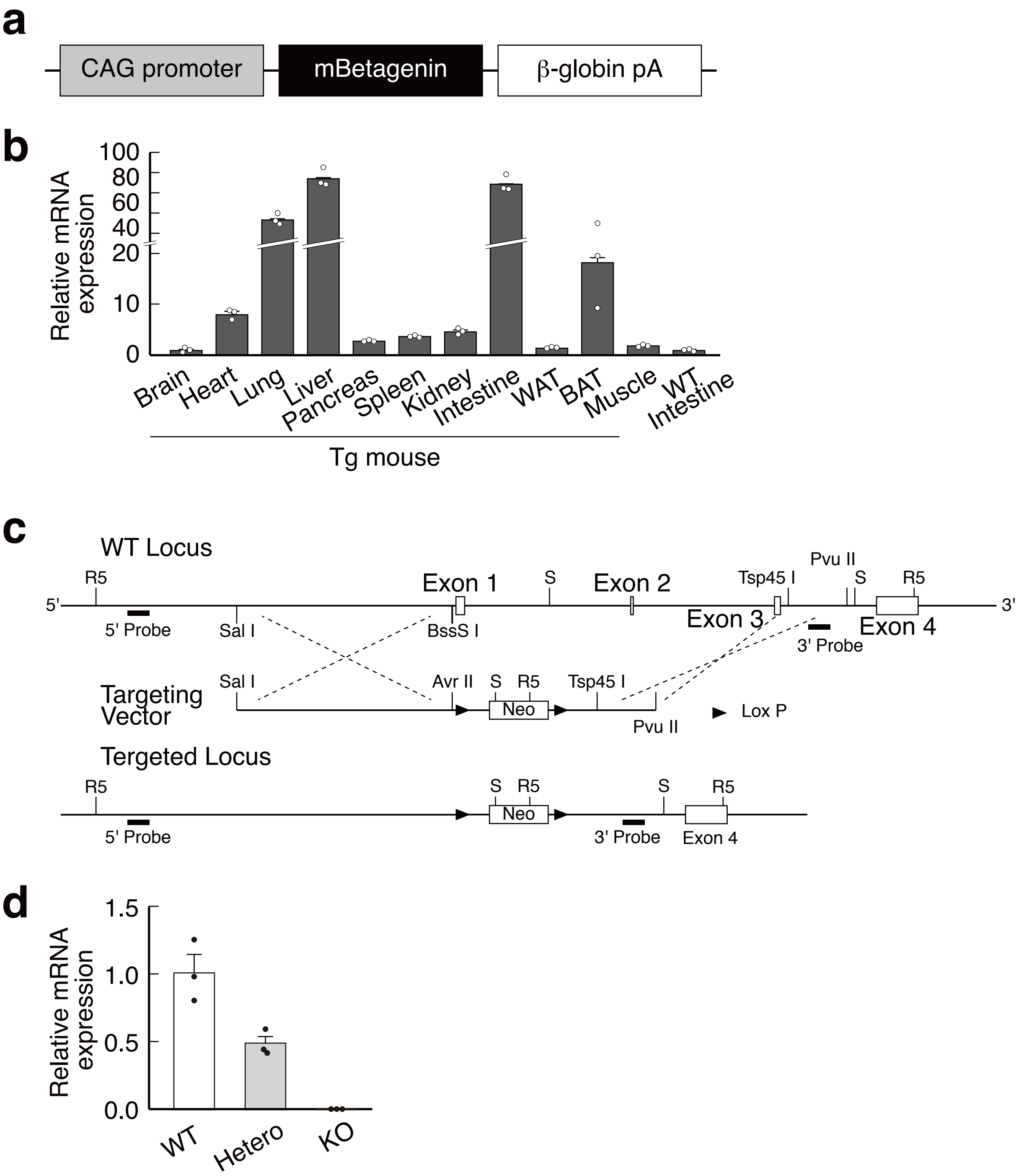

Supplementary Figure 3 | Design of the *Tm4sf20* transgenic (Tg) and knockout (KO) mice constructs. **(a)** The transgenic mice overexpressed *Tm4sf20* under the control of the CAG promoter. **(b)** QPCR analysis showing widespread expression of *Tm4sf20* in the tissues of transgenic mice. **(c)** Generation of *Tm4sf20*-KO mice. Neo, neomycin resistance cassette; R5, EcoRV; S, SalI. The location of the probe used for Southern blot analysis is denoted by the horizontal filled rectangle labeled “5′ and 3′ probe.” **(d)** *Tm4sf20* mRNA deletion in the intestine of the KO mice shown by qPCR.
